# Transference and the psychological interplay in AI-enhanced mental healthcare

**DOI:** 10.3389/fpsyt.2024.1460469

**Published:** 2024-08-19

**Authors:** Akhil P. Joseph, Anithamol Babu

**Affiliations:** ^1^ School of Social Work, Marian College Kuttikkanam Autonomous, Kuttikkanam, India; ^2^ Department of Sociology & Social Work, Christ (Deemed to be University), Bengaluru, India; ^3^ School of Social Work, Tata Institute of Social Sciences, Jalukbari, India

**Keywords:** transference, artificial intelligence, mental healthcare, chatbots, therapeutic alliance

Artificial intelligence revolutionizes mental healthcare by leveraging vast datasets to predict potential crises proactively, transforming treatment from reactive to preventive approaches. Remote patient monitoring tools and surveillance technologies enhance this transformation by providing real-time behavioral and symptom data, enabling immediate, personalized adjustments to treatment protocols, and ensuring adherence to therapeutic regimens (27). Examples of AI’s predictive capabilities include Mindstrong’s technology, which analyzes smartphone usage patterns to detect early signs of mood fluctuations (1, 2), and Tess, a virtual therapeutic assistant that uses natural language processing to provide real-time, personalized support to patients, extending care beyond traditional settings (3). These AI applications not only improve the accuracy of diagnostics and interventions but also democratize access to mental health services, making high-quality care accessible to larger populations and facilitating timely and tailored interventions uniquely suited to individual needs. This transformative impact of AI is reshaping mental health practices, presenting new opportunities and challenges that must be navigated with careful consideration of ethical implications and the human element in healthcare.

As AI profoundly transforms the therapeutic landscape, it redefines the pivotal dynamics of the therapist-patient relationship, a linchpin of successful mental health treatment (4). This technological evolution introduces sophisticated layers to the psychoanalytic concept of transference, where patients unconsciously project their feelings, expectations, or past experiences onto their therapist, a dynamic initially articulated by Sigmund Freud (5). Freud’s seminal work underscores how patients often transfer emotional responses from significant personal relationships into the therapeutic context, uncovering deeper, often unconscious psychological layers (6). Expanding on Freud, Carl Jung posited that transference is not merely the projection of past attitudes but represents a fundamental human inclination to replicate established relational patterns in new settings, suggesting that transference could also involve archetypal figures and motifs that are universally ingrained in the human psyche (7, 8). This is particularly significant in AI-enabled psychotherapy, where the apparatus of therapy—comprising the therapist, the patient, and the therapeutic environment—is reconstituted.

AI systems, designed based on principles from cognitive behavioral therapy and other therapeutic models, not only facilitate always-on availability and text-based interaction but also challenge traditional notions of transference by potentially invoking responses tied to the collective unconscious. Forming a therapeutic alliance is essential in psychotherapy, establishing trust and confidentiality is crucial for effective treatment, as noted by Edward Bordin (9), who identifies its core elements as agreeing on goals, assigning tasks, and developing bonds. However, in the context of AI-driven interactions, such as with therapeutic chatbots like Woebot, cannot form genuine emotional bonds, the goal shifts towards creating a Digital Therapeutic Alliance (DTA), a user-perceived connection where users engage with chatbots in a manner that aligns with therapeutic goals (10). This involves anthropomorphizing chatbots—attributing them with human-like qualities, which research shows can enhance trust and engagement (11). Patients often perceive these AI-driven therapy bots as empathetic and capable of understanding human emotions, a form of positive transference replicating aspects of a therapeutic alliance (11). However, this approach carries the risk of therapeutic misconception, where patients might misunderstand the capabilities and limitations of AI in therapy. While enhancing chatbot features can help bridge the gap left by a shortage of mental health professionals, it is crucial to maintain clear communication about the nature of AI interactions to ensure that the therapeutic use of AI remains effective and ethically sound (12).

Research evidence highlights how patients frequently project emotional responses onto AI systems during therapeutic engagements, a dynamic that mirrors traditional transference observed in human-to-human therapy. Therapy chatbots, such as Replika and Woebot, exemplify how users form deep emotional connections with AI systems, often treating these non-human entities as confidants and therapists (13). Users frequently report feelings of emotional safety and connection when interacting with these AIs, sharing personal, sensitive information much like they would with a human therapist. This behavior underscores a significant psychological engagement akin to what one might experience in traditional therapeutic settings. For instance, Replika, an AI designed to provide emotional support, has been noted for its ability to engage users in a manner that encourages them to express intimate thoughts and emotions that they might otherwise withhold (14). The AI’s consistent, non-judgmental interaction style not only mimics the therapeutic alliance found in human-to-human therapy but also creates a space for projecting familial and friendship roles onto the AI. Similarly, Woebot, which utilizes cognitive-behavioral therapy techniques, has shown how users often attribute the bot with a therapist’s acumen, reporting that the bot “understands” them (12). This perception facilitates a deeper therapeutic process as users transfer their expectations of empathy and support onto the AI, engaging more openly in psychological self-exploration. Furthermore, Ellie, a virtual therapist used primarily with veterans suffering from PTSD, leverages vocal and facial recognition technologies to gauge users’ emotional states. Veterans interacting with Ellie often treat the AI more like a peer than a clinical tool, disclosing traumatic experiences more freely due to the AI’s perceived non-judgmental nature (15). This form of transference, where the AI is perceived as an empathetic and understanding figure, can lead to significant breakthroughs in therapy, suggesting that projecting these feelings onto AI can have therapeutic benefits.

The novel form of transference in AI-driven therapy introduces unique challenges that demand a deep understanding of psychoanalytic theory and human psychology to manage effectively, ensuring that AI aids therapeutic breakthroughs without fostering harmful dependencies or reinforcing maladaptive behaviors (16). This intricate interplay between traditional psychoanalytic theories and modern AI applications requires meticulous attention to preserve patients’ therapeutic integrity and psychological well-being. While the constant availability of AI therapy bots offers a non-judgmental space for individuals with conditions such as depression and provides immediate coping strategies for those with anxiety disorders, this accessibility could foster an over-reliance on AI, potentially sidelining crucial human interactions and professional therapy vital for comprehensive treatment (17). Moreover, for individuals with personality disorders, particularly those characterized by intense emotional experiences and unstable interpersonal relationships, interactions with AI may lead to complex transference dynamics (18). These patients might experience negative transference, where they project hostile feelings and unrealistic expectations onto the AI. This could potentially escalate to scenarios where the AI’s responses are interpreted as personal slights or rejections, exacerbating the user’s condition rather than ameliorating it. For anxiety disorders, the predictable and consistent nature of AI interactions can serve as a stabilizing influence, reducing anxiety triggers and providing a non-threatening environment for therapy (19). For those with OCD, routine interaction with AI could inadvertently perpetuate compulsive behaviors, while individuals with eating disorders might receive reinforcement for obsessive patterns, deepening the disorder (20, 21). Patients with schizophrenia risk attributing real emotions to AI, potentially exacerbating delusional thinking (22). During manic or depressive phases of bipolar disorder, patients might overly engage with AI or become overly dependent on it for basic functioning (23). AI’s role in supporting individuals with substance use disorders or the elderly might lead to neglect of broader recovery support systems or social isolation from family and friends if AI becomes a substitute for human contact (24, 25). Thus, while AI can significantly augment therapeutic support, its integration into mental health practices necessitates carefully reevaluating how transference manifests and is handled in therapy to maximize therapeutic outcomes and maintain ethical standards in AI deployment.

The implications of transference in AI-driven mental health interventions necessitate rigorous oversight and thoughtful management. To effectively support therapeutic goals while avoiding pitfalls such as fostering dependency or reinforcing maladaptive behaviors, AI systems must be designed with a sophisticated understanding of both the underlying psychological dynamics and the ethical frameworks that govern their use. This calls for a high degree of technological innovation, particularly in developing emotional AI that can sensitively interpret and respond to human emotions in a therapeutic context (26). As AI becomes more embedded in mental healthcare, mastering the management of transference phenomena is crucial for maximizing therapeutic outcomes and safeguarding ethical standards in deploying these advanced technologies.

Integrating an understanding of transference into AI-driven mental health tools brings forth significant ethical and practical challenges, highlighting concerns over the potential manipulation of emotional transference for commercial benefits or boosting user engagement. Such manipulations exploit the human tendency to form attachments and project emotions, which could exacerbate mental health issues and erode trust in technological healthcare solutions, skewing the focus from therapeutic value to business metrics (26, 27). Furthermore, if AI systems become too adept at eliciting and managing transference, it might blur the lines between genuine therapeutic relationships and artificial interactions, potentially leading to detrimental attachments to these systems. This scenario risks replacing essential human contact and genuine emotional connections with superficial interactions, depriving individuals of the complex interactions vital for comprehensive mental health. To mitigate these risks, the development of AI in mental health care must adhere to stringent ethical guidelines prioritizing patient welfare, advocating for transparency, informed consent, privacy protection, and the minimization of dependency on technology. Moreover, regulatory frameworks need to be established to monitor and evaluate the impact of AI applications on patient health and privacy, promoting an ethical approach to mental healthcare that respects and enhances human dignity and well-being. Such measures are crucial to prevent AI from exploiting emotional transference for commercial purposes, which poses a profound ethical concern as outlined by recent discussions in the field (28, 29).

On a practical level, developing AI technologies that can recognize and appropriately manage transference presents a formidable challenge. Current AI systems, while increasingly sophisticated, generally need more emotional intelligence to fully understand and respond to the complex dynamics of human psychological processes. For AI to effectively manage transference, it must be capable of interpreting subtle cues and nuances in human behavior and emotional expression akin to those understood by a skilled human therapist (26). This requires advances in natural language processing and emotional recognition technologies and a framework that allows AI to make nuanced decisions regarding an individual’s emotional and psychological needs. Besides, AI systems must be designed to identify instances of transference and respond in supportive and therapeutic ways without fostering dependency or reinforcing maladaptive behaviors. This involves the creation of AI that is not only technically proficient but also deeply integrated with psychological theories and therapeutic practices. AI developers and mental health professionals must collaborate closely to ensure that AI systems are informed by a thorough understanding of transference and other psychoanalytic concepts. Moreover, there is a need for continuous monitoring and evaluation of AI interactions in mental health settings to ensure that these technologies are used responsibly and effectively. Implementing regulatory frameworks and ethical guidelines will be essential to govern the development and deployment of AI in mental health care. These measures should ensure that AI systems enhance therapeutic outcomes, respect patient autonomy, and remain sensitive to the complex ethical implications of their use in mental health contexts.

Transference in AI interactions represents a pivotal intersection of technology, psychology, and psychiatry within mental healthcare. Understanding and adeptly managing this phenomenon is crucial for developing AI tools that are not only technologically advanced but also ethically attuned and psychologically sensitive. As AI continues to evolve, our methodologies for integrating it into therapeutic settings must also progress, ensuring that these tools enhance traditional psychological therapies. Addressing the complexities of transference in AI-enhanced mental healthcare necessitates robust interdisciplinary collaboration among psychologists, psychiatrists, AI developers, and ethicists to craft applications that respect and promote human psychological well-being. Moreover, incorporating a global perspective is essential, as cultural differences can significantly influence the reception and effectiveness of AI in mental healthcare. There is also a pressing need to analyze therapists’ perspectives on AI’s role in therapy to guide the development of tools that support, rather than supplant, the therapeutic relationship. Future research should focus on developing AI systems that adapt to feedback from therapeutic interactions, leveraging machine learning to refine responses based on diverse contexts and outcomes, and ensuring that AI development in mental healthcare is both innovative and inclusive, thereby enhancing care quality and maintaining ethical integrity.
